# Health-Related Quality of Life Among Pregnant Women With Pre-pregnancy Smoking and Smoking Cessation During Pregnancy in China: National Cross-sectional Study

**DOI:** 10.2196/29718

**Published:** 2022-01-24

**Authors:** Kadi Hu, Shiqian Zou, Casper JP Zhang, Huailiang Wu, Babatunde Akinwunmi, Zilian Wang, Wai-Kit Ming

**Affiliations:** 1 International School Jinan University Guangzhou China; 2 School of Public Health Li Ka Shing Faculty of Medicine The University of Hong Kong Hong Kong China; 3 Department of Obstetrics and Gynecology Brigham and Women's Hospital Harvard Medical School Boston, MA United States; 4 Center for Genomic Medicine Massachusetts General Hospital Harvard Medical School Boston, MA United States; 5 Department of Obstetrics and Gynaecology The First Affiliated Hospital of Sun Yat-sen University Guangzhou China; 6 Department of Infectious Diseases and Public Health Jockey Club College of Veterinary Medicine and Life Sciences City University of Hong Kong Hong Kong China; 7 School of Public Policy and Management Tsinghua University Beijing China

**Keywords:** health-related quality of life, pregnant women, smoking status, pre-pregnancy smoking

## Abstract

**Background:**

Previous studies have hardly explored the influence of pre-pregnancy smoking and smoking cessation during pregnancy on the health-related quality of life (HRQoL) of pregnant women, which is a topic that need to be addressed. In addition, pregnant women in China constitute a big population in the largest developing country of the world and cannot be neglected.

**Objective:**

This study aims to evaluate the HRQoL of pregnant women in China with different smoking statuses and further estimate the association between pre-pregnancy smoking, smoking cessation, and the HRQoL.

**Methods:**

A nationwide cross-sectional study was conducted to determine the association between different smoking statuses (smoking currently, quit smoking, never smoking) and the HRQoL in pregnant women across mainland China. A web-based questionnaire was delivered through the Banmi Online Maternity School platform, including questions about demographics, smoking status, and the HRQoL. EuroQoL Group’s 5-dimension 5-level (EQ-5D-5L) scale with EuroQoL Group’s visual analog scale (EQ-VAS) was used for measuring the HRQoL. Ethical approval was granted by the institutional review board of the First Affiliated Hospital of Sun Yat-sen University (ICE-2017-296).

**Results:**

From August to September 2019, a total of 16,483 participants from 31 provinces were included, of which 93 (0.56%) were smokers, 731 (4.43%) were ex-smokers, and 15,659 (95%) were nonsmokers. Nonsmokers had the highest EQ-VAS score (mean 84.49, SD 14.84), smokers had the lowest EQ-VAS score (mean 77.38, SD 21.99), and the EQ-VAS score for ex-smokers was in between (mean 81.04, SD 17.68). A significant difference in EQ-VAS scores was detected between nonsmokers and ex-smokers (*P*<.001), which indicated that pre-pregnancy smoking does have a negative impact on the HRQoL (EQ-VAS) of pregnant women. Compared with nonsmokers, ex-smokers suffered from more anxiety/depression problems (*P*=.001, odds ratio [OR] 1.29, 95% CI 1.12-1.50). Among ex-smokers, the increased cigarette consumption was associated with a lower EQ-5D index (*P*=.007) and EQ-VAS score (*P*=.01) of pregnant women. Compared to smokers, no significant difference was found in the ex-smokers’ EQ-5D index and EQ-VAS score (*P*=.33).

**Conclusions:**

Smoking history is associated with a lower HRQoL in pregnant Chinese women. Pre-pregnancy smoking is related to a lower HRQoL (EQ-VAS) and a higher incidence of depression/anxiety problems. Smoking cessation during pregnancy does not significantly improve the HRQoL of pregnant Chinese women. Among ex-smokers, the more cigarettes they smoke, the lower HRQoL they have during pregnancy. We suggest that the Chinese government should strengthen the education on quitting smoking and avoiding second-hand smoke for women who have pregnancy plans and their family members.

## Introduction

Active smoking increases the risk of developing chronic diseases and malignancy, such as chronic obstructive pulmonary disease and lung cancer [1]. Until 2019, there were more than 1 million tobacco-caused deaths in China, and the hazards are expected to increase substantially in the next few decades [2-4]. Smoking has also been proven to impair reproductive function, and during pregnancy, it was identified as a risk factor for terrible clinical outcomes, such as stillbirth and abortion [5,6]. In China, although most of the women who smoke quit smoking when they are pregnant, the prevalence of smoking among pregnant women still reached 3.8% [7], which is higher than that of women in general (2.4%) [8]. In addition, the prevalence of smoking in women younger than 40 years old, who are at reproductive age, has increased significantly in recent years [7]. Therefore, the pregnant Chinese women’s health-related quality of life (HRQoL) and its relationship to smoking needs to be explored.

The World Health Organization reported that tobacco use is a major risk factor for cardiovascular diseases, respiratory diseases, and cancers [9]. At the same time, nicotine withdrawal causes mental symptoms, including insomnia, anxiety, depression, and anhedonia [10]. In the general population, smoking cessation leads to a higher perceived quality of life [11]. However, among pregnant women, the health status of those quitting smoking after pregnancy was still worse than that of nonsmokers [12], and smoking-related health consequences occurred in most of the pregnant ex-smokers, which affected their somatic health [13]. A previous study addressed the effect of smoking before pregnancy [14], but it merely included smoking during the 3 months before conception as preconception smoking and did not explore the impact of pre-pregnancy on a wider circle of mental health. Furthermore, although the effect of smoking cessation has been explored in the general population [15], the exact effect of smoking cessation during pregnancy on the health status of pregnant women is still unclear. Therefore, it is necessary to evaluate the impact of pre-pregnancy smoking and smoking cessation during pregnancy on both physical health and mental health of pregnant women, especially in China, which is the largest country in the world.

The HRQoL is a multidimensional indicator for measuring people’s physical, mental, emotional, and social health states in their lives over time. The HRQoL not only benefits the health perception at the individual level but also enables health agencies in legislation, community health planning, and business health projects [16]. The HRQoL of women who quit smoking during pregnancy can be used as an outcome indicator, which can facilitate the progression in pre-pregnancy smoking and smoking cessation management. Moreover, prevention is as important as cure in medicine, and knowing the impact of pre-pregnancy smoking and smoking cessation during pregnancy can help pregnant women prevent smoking-associated complications [17].

Considering its importance, we aim to explore the effect of pre-pregnancy smoking and smoking cessation during pregnancy on pregnant women’s HRQoL in mainland China and compare the effects of pre-pregnancy smoking on pregnant women’s HRQoL (5 health dimensions). Additionally, this study also explored the relationship between the number of cigarettes consumed and the HRQoL of pregnant women in mainland China.

## Methods

### Study Design

A nationwide cross-sectional study was performed to investigate pregnant women’s HRQoL using a self-administrative questionnaire across mainland China. The questionnaire was designed based on the Global Tobacco Surveillance System and EuroQoL Group’s 5-dimension (EQ-5D) questionnaire [18], which is a group of instrumental questionnaires to assess people’s HRQoL, make cost-efficiency calculations, and evaluate economic issues in the public health field [19]. It has been proven that the Chinese version of the EQ-5D index can effectively measure the HRQoL of pregnant women [20]. In EQ-5D questionnaires, EuroQol Group’s 5-dimension 5-level (EQ-5D-5L) scale and EuroQoL Group’s visual analog scale (EQ-VAS) are more reliable and were used to measure the HRQoL in this study. The questionnaire includes a total of 10 fixed questions and 2 adaptive scales on 1 page, including demographics questions, smoking status questions, the EQ-5D-5L scale, and the EQ-VAS. A completeness check was applied, and participants were not allowed to submit the questionnaire until they responded to all the questions. Participants were not able to review or change their answers after submission.

Ethical approval was granted by the institutional review board of the First Affiliated Hospital of Sun Yat-sen University (ICE-2017-296). All procedures were conducted following the Declaration of Helsinki. All participants signed the informed consent documents before participation in this study.

### Study Population and Recruitment

The web-based questionnaire was distributed through a national online platform (Banmi Online Maternity School) from August to September 2019. The Banmi Online Maternity School is a free platform that provides pregnancy knowledge for all internet users and serves more than 1 million users across China. The research group members of the Banmi Online Maternity School were the investigators. We advertised the survey with the wording “For providing you with more specific gestational health knowledge, we invite you to participate in this survey,” and no incentive was provided. A total of 16,811 questionnaires from pregnant women aged from 16 to 60 years were included, and 328 (1.95%) of them were excluded due to the living location not being mainland China. The final sample comprised 16,483 pregnant women from mainland China. According to the standards of the Chinese Center for Disease Control and Prevention, the research was performed in 7 administrative regions of mainland China: (1) the Northeast (Heilongjiang, Jilin, and Liaoning), (2) the North (Beijing, Tianjin, Hebei, Shanxi, and Inner Mongolia), (3) Central (Hubei, Hunan, and Henan), (4) the East (Shanghai, Shandong, Jiangsu, Anhui, Jiangxi, Zhejiang, and Fujian), (5) the South (Guangdong, Guangxi, and Hainan), (6) the Northwest (Shanxi, Gansu, Ningxia, and Xinjiang), and (7) the Southwest (Chongqing, Sichuan, Guizhou, Yunnan, and Tibet).

### Variables

Participants’ sociodemographic information, including age, gestational age (weeks), address (provinces and cities), disposable income, smoking status, amount of cigarette consumption, smoking status of the spouse, and smoking duration (years), were collected. Previous studies have reported that maternal age, gestational age, and income level are related to people’s HRQoL [21]. The independent variables in our study were the smoking status and cigarette consumption of pregnant women. To determine the smoking status, participants were provided with the following options: (1) currently smoking, (2) smoking only before pregnancy, and (3) never smoked. They were classified into (1) smokers, (2) ex-smokers, and (3) nonsmokers. Smokers and ex-smokers were further asked for the number of cigarettes they consumed per day and classified as mild (1-9 cigarettes), moderate (10-19 cigarettes), and heavy (>20 cigarettes) smokers [22].

### Measurement

We use the EQ-5D instrument, which consists of the EQ-5D-5L scale and the EQ-VAS, to evaluate the HRQoL of pregnant women. The EQ-5D-5L scale assesses 5 dimensions: mobility, self-care, usual activity, pain/discomfort, and anxiety/depression. Further, each dimension is addressed by 5 levels: (1) none, (2) slight problem, (3) moderate problem, (4) severe problem, and (5) extreme problem/unable. All dimension levels were converted into 1, 2, 3, 4, or 5 in the given order. Next, an EQ-5D index for each participant was calculated using the EQ-5D-5L Crosswalk Index Value Calculator. The possible maximal EQ-5D index is in the range of –0.224-1, where 1 indicates the highest health status, 0 represents death, and negative indices indicate the health status considered worse than death [20,23,24]. The EQ-VAS was presented as a calibrated vertical line from 0 (worst) to 100 (best) [25]. Participants were asked to mark on the vertical line of the VAS based on their own perceptions of their health status. Generally, both a higher EQ-5D index and a higher EQ-VAS score indicate a better HRQoL.

### Statistical Analysis

Data analysis was performed using STATA/SE version 14.0 for Windows (College Station, TX, USA). Normally distributed continuous variables were described using means and SDs. Nonnormal variables were presented as the median, and categorical variables were described using counts and percentages. Demographic data, including age, gestational age, address, smoking status, spouse’s smoking status, EQ-5D index, and EQ-VAS score, were included. The EQ-5D index and the EQ-VAS score were the outcome variables, and they were not normally distributed. A 1-way ANOVA test was performed to compare the continuous variables and analyze their variances. The Bartlett test was used to determine unequal variances. The Tamhane T2 method was used for pairwise comparison tests of EQ-VAS scores between groups, and the chi-square test performed to analyze the proportion of spouse smoking among groups. To estimate the relationship between independent variables (demographics) and dependent variables (EQ-5D index and EQ-VAS score), we also ran an ordinary least squares regression, which minimized the sum of the squared residuals to obtain adjusted values of the dependent variables. For nonsmokers and smokers, an ordered logistic regression with odds ratios (ORs) and 95% CIs was run to assess the effects of independent factors on each dimension of EQ-5D indices. In the ordered logistic regression analysis, pre-pregnancy smoking was a dichotomous variable consisting of no smoking behavior (nonsmoker) and quitting smoking during pregnancy (ex-smoker). All tests were 2-sided, and *P*<.05 was considered statistically significant [20,26].

### Bias

Because the HRQoL is related to individuals’ perception of their position of life in the context of the culture and value systems in which they live, transnational culture differences will have an obvious impact on the HRQoL. Our study, which was conducted in China, avoided this potential difference [19]. We performed data desensitization before data cleaning and analysis. Although smoking status diversifies with educational levels and geographic factors, the large number of participants from many different areas in China in this study likely minimized selection bias. Since a completeness check was applied in the questionnaire-collecting process, the study has no nonresponse bias. Moreover, because only pregnant women who took part in the maternity school received questionnaires, the study has no ascertainment bias. The EQ-5D-5L scale and the EQ-VAS are subjective measurements of pregnant women’s HRQoL. Therefore, self-reported bias is the main bias in this study.

## Results

### Participants

**Figure 1:**
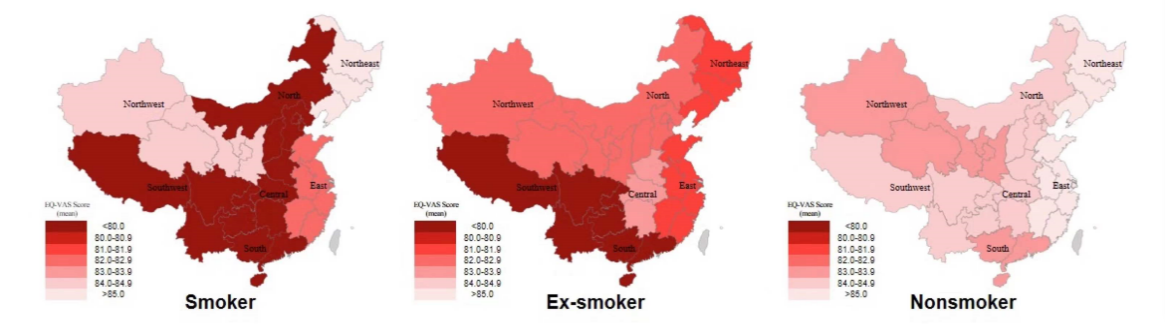
Geographical distribution of pregnant women’s EQ-VAS scores of (A) smokers, (B) ex-smokers, and (C) nonsmokers across the 7 administrative regions in mainland China. EQ-VAS: EuroQoL Group’s visual analog scale.

From August to September 2019, a total of 16,483 participants from 30 provinces were included (Table 1 and Figure 1). The participants in our study were not characteristically different from the general pregnant women in China, except that those who had no access to the internet were not included.

**Table 1:** Demographics, EQ-5D^a^ indices, and EQ-VAS^b^ scores of pregnant women with different smoking statuses (N=16,483).

Characteristic	Smoker (n=93)	Ex-smoker (n=731)	Nonsmoker (n=15,659)	*P* value
Age (years), mean (SD)	26.45 (5.43)	26.18 (5.52)	28.25 (4.91)	<.001^c^
Gestational age (weeks), mean (SD)	21.17 (8.87)	20.50 (9.55)	21.12 (9.09)	.19
Spouse smoking, n (%)	87 (94)	607 (83.0)	8758 (56.0)	<.001^c^
Disposable income (CN ¥^d^), mean (SD)	28,589.01 (8680.59)	28,247.57 (8126.92)	29,978.01 (9321.93)	<.001^c^
Smoking duration (years), mean (SD)	19.53 (7.26)	20.37 (5.84)	—^e^	.20
EQ-5D index, mean (SD)	0.82 (0.14)	0.80 (0.12)	0.80 (0.13)	.16
EQ-VAS score, mean (SD)	77.38 (21.99)	81.04 (17.68)	84.49 (14.84)	<.001^c^

^a^EQ-5D: EuroQol Group’s 5-dimension.

^b^EQ-VAS: EuroQoL Group’s visual analog scale.

^c^*P*<.05.

^d^A currency exchange rate of CN ¥1= US $0.13971 was applicable per OANDA Rates in September 1, 2019.

^e^No result.

### General Characteristics of Participants

Of the 16,483 participants, 93 (0.56%) were smokers, 731 (4.43%) were ex-smokers, and 15,659 (95%) were nonsmokers (Table 1). For the smoker group, the mean (SD) was 26.45 (5.43) years for age, 21.17 (8.87) weeks for gestational age, and 19.53 (7.26) years for smoking duration. Ex-smokers had an average gestational age of 20.50 (9.55) weeks and a smoking duration of 20.37 (5.84). Nonsmokers had an average gestational age of 21.12 (9.09) weeks.

**Figure 2:**
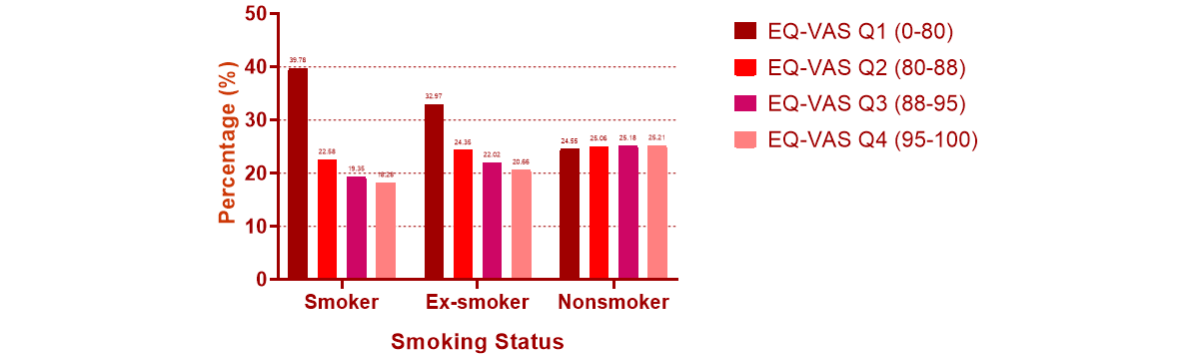
EQ-VAS distribution according to smoking status and IQR. EQ-VAS: EuroQoL Group’s visual analog scale.

Smokers, ex-smokers, and nonsmokers had an EQ-5D index of 0.82 (0.14), 0.80 (0.12), and 0.80 (0.13), respectively (Table 1). The EQ-VAS score was found to be statistically different among smokers (mean 77.38, SD 21.99), ex-smokers (mean 81.04, SD 17.68), and nonsmokers (mean 84.49, SD 14.84; all *P*<.001). Figures 1 and 2 reveal the EQ-VAS scores’ distribution among pregnant Chinese women according to their geographic location and smoking status. Pregnant women who were nonsmokers had the highest, while the smokers had the lowest EQ-VAS scores. For nonsmokers, those living in Northeast and East China tended to have higher EQ-VAS scores, and those living in Northwest and South China had lower EQ-VAS scores. For smokers and ex-smokers, pregnant women living in Southwest and South China tended to have lower EQ-VAS scores. A significant difference was found in age, spouse smoking rate, and disposable income (all *P*<.001). Therefore, age, spouse smoking rate, and disposable income were adjusted in the analysis. After adjustment, the EQ-5D index was still not statistically different (*P*=.82), and the EQ-VAS score was still statistically different (*P*<.001) between pregnant women who were smokers, ex-smokers, and nonsmokers (Table 2). No significant difference was found in gestational age (*P*=.19) and the EQ-5D index (*P*=.16) among the 3 groups, and smoking duration showed no significant difference between smokers and ex-smokers (*P*=.20).

**Table 2:** EQ-5D^a^ index and EQ-VAS^b^ scores among smokers, ex-smokers, and nonsmokers (N=16,483).

Smoking status		Unadjusted value, mean (SD)	Age, per capita disposable income, and spouse smoking status adjusted, mean (SD)
**EQ-5D index**			
	Smoker	0.82 (0.14)	0.80 (0.00)
	Ex-smoker	0.8 (0.12)	0.80 (0.00)
	Nonsmoker	0.8 (0.13)	0.80 (0.00)
	*P* value	0.16	0.82
**EQ-VAS**			
	Smoker	77.38 (21.99)	78.08 (0.13)
	Ex-smoker	81.04 (17.68)	80.86 (0.05)
	Nonsmoker	84.49 (14.84)	84.49 (0.01)
	*P* value	<.001^c^	<.001^c^

^a^EQ-5D: EuroQol Group’s 5-dimension.

^b^EQ-VAS: EuroQoL Group’s visual analog scale.

^c^*P*<.05.

### EQ-VAS Scores for Different Smoking Statuses

Table 3 presents the multicomparison of EQ-VAS scores between groups after the unequal variance test (*P*<.001). Significant differences were observed in the EQ-VAS scores, with of nonsmokers having the highest score of 84.49 (14.84). Specifically, their EQ-VAS scores were higher than those of smokers (mean difference=7.11, *P*=.01, 95% CI 1.56-12.66) and ex-smokers (mean difference=3.45, *P*<.001). However, no significant difference was found between smokers and ex-smokers (*P*=.33).

**Table 3:** Pairwise comparisons of pregnant women’s smoking status and EQ-VAS^a^ score between groups.

Pairwise groups	Mean difference	SE	95% CI	*P* value
Smoker vs ex-smoker	3.66	2.37	–2.09 to 9.41	.33
Smoker vs nonsmoker	7.11	2.28	1.56-12.66	.007^b^
Ex-smoker vs nonsmoker	3.45	0.66	1.86-5.04	<.001^b^

^a^EQ-VAS: EuroQoL Group’s visual analog scale.

^b^*P*<.05.

### Comparison of the Smoking Status in the 5 Dimensions of EQ-5D Index

Table 4 shows the frequency of the EQ-5D index in 5 dimensions by smoking status. In total, 3717 of 16,483 (22.55%) pregnant women reported health-related problems (levels 2-5) in the mobility dimension, 971 (5.89%) reported self-care problems, 3337 (20.25%) reported usual activity problems, 9298 (56.41%) reported pain and discomfort problems, and 8487 (51.49%) reported anxiety/depression problems. Results revealed that the main limited health dimension for pregnant women is pain/discomfort. According to the results in Table 4, the main health problem for ex-smokers and nonsmokers was pain/discomfort; among them, 429 of 731 ex-smokers (58.7%) and 8763 of 15,659 nonsmokers (55.9%) reporting related problems. As the main health problem, depression/anxiety was reported by 52 of 93 smokers (56%).

**Table 4:** Frequency (%) of the EQ-5D^a^ index of pregnant women with different smoking statuses (N=16,483).

EQ-5D dimension			Smoker, n (%)		Ex-smoker, n (%)		Nonsmoker, n (%)	Total, n (%)
**Mobility**								
	Level 1	75 (81)		575 (78.7)		12,116 (77.4)		12,766 (77.45)
	Level 2	18 (19)		125 (17.1)		2969 (19.0)		3112 (18.88)
	Level 3	0		27 (3.7)		460 (2.9)		487 (2.95)
	Level 4	0		4 (0.6)		58 (0.4)		62 (0.38)
	Level 5	0		0 (0.0)		56 (0.4)		56 (0.34)
**Self-care**								
	Level 1	87 (94)		689 (94.3)		14,736 (94.1)		15,512 (94.11)
	Level 2	4 (4)		38 (5.2)		843 (5.4)		885 (5.37)
	Level 3	0		1 (0.1)		58 (0.4)		59 (0.36)
	Level 4	1 (1)		2 (0.3)		10 (0.1)		13 (0.08)
	Level 5	1 (1)		1 (0.1)		12 (0.1)		14 (0.07)
**Usual activity**								
	Level 1	80 (86)		606 (82.9)		12,460 (79.6)		13,146 (79.75)
	Level 2	10 (11)		116 (15.9)		2875 (18.4)		3001 (18.21)
	Level 3	0		7 (1.0)		245 (1.6)		252 (1.53)
	Level 4	1 (1)		1 (0.1)		27 (0.2)		29 (0.18)
	Level 5	2 (2)		1 (0.1)		52 (0.3)		55 (0.33)
**Pain/discomfort**								
	Level 1	47 (51)		302 (41.3)		6836 (43.7)		7185 (43.59)
	Level 2	42 (45)		385 (52.7)		8111 (51.8)		8538 (51.79)
	Level 3	3 (3)		33 (4.5)		627 (4.0)		663 (4.02)
	Level 4	1 (1)		10 (1.4)		68 (0.4)		79 (0.48)
	Level 5	0		1 (0.1)		17 (0.1)		18 (0.11)
**Depression/anxiety**								
	Level 1	39 (41)		309 (42.3)		7648 (48.8)		7996 (48.51)
	Level 2	42 (45)		346 (47.3)		7086 (45.3)		7474 (45.34)
	Level 3	11 (12)		56 (7.7)		750 (4.8)		817 (4.96)
	Level 4	0		14 (1.9)		129 (0.8)		143 (0.87)
	Level 5	1 (1)		6 (0.8)		46 (0.3)		53 (0.32)

^a^EQ-5D: EuroQol Group’s 5-dimension.

Table 5 reveals the impact of risk factors on the 5 dimensions of EQ-5D-5L scale. Results indicated that increasing age and gestational age are positively related to mobility (both *P*<.001, OR 1.02, 95% CI 1.01-1.03; OR 1.04, 95% CI 1.03-1.04, respectively) and usual activity problems (*P*=.01, OR 1.01, 95% CI 1.00-1.02; *P*<.001, OR 1.03, 95% CI 1.03-1.04, respectively). Increasing age was negatively related to self-care (*P*=.03, OR 0.98, 95% CI 0.97-1.00), pain/discomfort (*P*<.001, OR 0.98, 95% CI 0.97-0.99), and anxiety/depression problems (*P*<.001, OR 0.98, 95% CI 0.97-0.98), while increasing gestational age was negatively related to them (*P*<.001, OR 1.08, 95% CI 1.07-1.09; *P*<.001, OR 1.02, 95% CI 1.02-1.02; and *P*<.001, OR 1.01, 95% CI 1.00-1.01, respectively). Spouse smoking (yes) was negatively related to self-care (*P*<.001, OR 0.79, 95% CI 0.69-0.90) and usual activity problems (*P*=.001, OR 0.88, 95% CI 0.81-1.95) but positively related to anxiety/depression problems (*P*=.01, OR 1.09, 95% CI 1.03-1.16). No correlation was found between disposable income and any health dimension. Pre-pregnancy smoking (yes) had a significant positive relationship with anxiety/depression problems (*P*=.001, OR 1.29, 95% CI 1.12-1.50).

**Table 5:** Ordered logistic regression analysis for each dimension in the EQ-5D^a^ index of nonsmokers and ex-smokers (n=16,390).

Dimension		OR^b^ (95% CI)		*P* value
**Mobility**				
	Age	1.02 (1.01-1.03)	<.001^c^	
	Spouse smoking	1.00 (0.93-1.08)	.93	
	Disposable income	1.00 (1.00-1.00)	.002^c^	
	Gestational age	1.04 (1.03-1.04)	<.001^c^	
	Pre-pregnancy smoking^d^	0.99 (0.82-1.18)	.88	
**Self-care**				
	Age	0.98 (0.97-1.00)	.02^c^	
	Spouse smoking	0.79 (0.69-0.90)	<.001^c^	
	Disposable income	1.00 (1.00-1.00)	.15	
	Gestational age	1.08 (1.07-1.09)	<.001^c^	
	Pre-pregnancy smoking^d^	1.01 (0.73-1.41)	.93	
**Usual activity**				
	Age	1.01 (1.00-1.02)	.01^c^	
	Spouse smoking	0.88 (0.81-1.95)	.001^c^	
	Disposable income	1.00 (1.00-1.00)	0.37	
	Gestational age	1.03 (1.03-1.04)	<.001^c^	
	Pre-pregnancy smoking^d^	0.86 (0.70-1.05)	.13	
**Pain/discomfort**				
	Age	0.98 (0.97-0.99)	<.001^c^	
	Spouse smoking	1.00 (0.94-1.06)	.89	
	Disposable income	1.00 (1.00-1.00)	.001^c^	
	Gestational age	1.02 (1.02-1.02)	<.001^c^	
	Pre-pregnancy smoking^d^	1.09 (0.94-1.27)	.24	
**Anxiety/depression**				
	Age	0.98 (0.97-0.98)	<.001^c^	
	Spouse smoking	1.09 (1.03-1.16)	.006^c^	
	Disposable income	1.00 (1.00-1.00)	.001^c^	
	Gestational age	1.01 (1.00-1.01)	<.001^c^	
	Pre-pregnancy smoking^d^	1.29 (1.12-1.50)	.001^c^	

^a^EQ-5D: EuroQol Group’s 5-dimension.

^b^OR: odds ratio.

^c^*P*<.05.

^d^Pre-pregnancy smoking is a dichotomous variable consisting of no smoking behavior (nonsmoker, represented as 0) and quitting smoking during pregnancy (ex-smoker, represented as 1).

### Amount of Cigarette Consumption and HRQoL

Table 6 shows the association of the EQ-5D index and EQ-VAS score with the amount of cigarette consumption per day among ex-smokers. Pregnant women who were ex-smokers were divided into 3 groups based on the amount of cigarette smoking. Significant differences across the 3 groups were found in both the EQ-5D index (*P*=.007) and the EQ-VAS score (*P*=.01). Moreover, both the EQ-5D index (mean 0.73, SD 0.16) and the EQ-VAS score (mean 67.93, SD 22.79) of heavy smokers were lower than those of moderate smokers (mean 0.77, SD 0.11 and mean 79.38, SD 17.82, respectively), while mild smokers had the highest EQ-5D index (mean 0.80, SD 0.12) and EQ-VAS score (mean 81.53, SD 17.45).

**Table 6:** EQ-5D^a^ index and EQ-VAS^b^ score for pregnant ex-smokers (n=731).

	Mild smoker (n=638)	Moderate smoker (n=79)	Heavy smoker (n=14)	*P* value^c^
EQ-5D index, mean (SD)	0.80 (0.12)	0.77 (0.10)	0.73 (0.16)	.007
EQ-VAS, mean (SD)	81.53 (17.45)	79.38 (17.82)	67.93 (22.79)	.01

^a^EQ-5D: EuroQol Group’s 5-dimension.

^b^EQ-VAS: EuroQoL Group’s visual analog scale.

^c^*P*<.05.

## Discussion

### Principal Findings

Smoking history (whether before or during pregnancy) is related to a worse HRQoL for of pregnant women. Smoking cessation during pregnancy does not significantly improve pregnant women’s HRQoL. Pre-pregnancy smoking is related to a worse HRQoL (EQ-VAS score) and a higher risk of anxiety/depression problems. In mainland China, pregnant smokers tend to have partners who are smokers. Moreover, the more cigarettes pregnant ex-smokers consume per day, the lower their HRQoL.

### Limitations

We found a few limitations of our study. First, the study was conducted online, so pregnant women without access to the internet were not included. Second, this study divided participants only into 3 groups according to their smoking history. Although we adjusted the impact of age, spouse smoking rate, and disposable income on the HRQoL of participants, there are still many other endogenous factors that can affect pregnant women’s HRQoL (eg, years of schooling, body mass index, chronic disease, abortion history) [27]. Future studies in this field should consider more factors that can affect pregnant women’s HRQoL.

### Comparison With Prior Works

Our results revealed that pregnant women with a smoking history, whether ex-smokers or smokers, have a lower HRQoL (EQ-VAS score) compared to nonsmokers. This result is similar to previous studies that reported that among women, smokers have a lower HRQoL compared to never-smokers [14,28]. A possible reason for this might be the harmful effect of pregnant women’s smoking experience on their physical health, especially trachea and lung health [29]. Another possible explanation is the spouse smoking percentage, in which the ex-smoker group had a higher spouse smoking rate than the nonsmoker group and a lower spouse smoking rate than the smoker group. Pregnant women are exposed to a second-hand smoke environment if their spouses smoke, which results in detrimental effects on the pregnant women and can lead to asthma, lung cancer, ischemic heart disease, etc [30].

The significant difference between EQ-VAS scores of ex-smokers and nonsmokers revealed the negative effect of pre-pregnancy smoking on pregnant women’s HRQoL in China. Before our study, few studies have addressed the effect of pre-pregnancy smoking on pregnant women’s health. However, the only study in this field included only 3 months prior to conception as pre-pregnancy smoking, did not explore a wide range of mental issues, and reported that women who smoked during the 3 months prior to conception were more likely to report poor vitality than nonsmokers [31], which was similar to our results. However, our study provided information. Our study revealed that pre-pregnancy smoking is related to a worse HRQoL among pregnant women and showed a significant correlation between pre-pregnancy smoking and the anxiety/depression dimension. A possible reason for the overall health decline due to pre-pregnancy smoking is the health reduction caused by smoking, including a higher risk of cancer, heart disease, and stroke [32]. The significant negative impact of pre-pregnancy smoking on the anxiety/depression dimension might be due to the brain damage linked to smoking [33].

The insignificant EQ-VAS score difference between smokers and ex-smokers revealed that smoking cessation cannot significantly improve the HRQoL of pregnant women in China. This was similar to a previous study that investigated people but not pregnant women and concluded that quitting smoking alone does not improve an individual’s HRQoL [28].

At the same time, the average EQ-5D index of pregnant women who were ex-smokers was not significantly different from that of other groups. Further analysis of the EQ-5D index revealed that the major problems for pregnant women in China are pain/discomfort problems. For smokers and nonsmokers, the anxiety/depression limitation is the most bothersome problem. Therefore, future policy planning in China should consider pain/discomfort care and mental health care during pregnancy.

Age was related to worsening mobility, which might be due to the decreasing mobility as people get older [34]. Gestational age was also related to worsening mobility, which can be explained by the limited movement for the heavier weight of pregnant women [35]. For the self-care dimension, age and gestational age were related to less self-care. A possible explanation for this is that older pregnant women or pregnant women of advanced gestational age tend to have more pregnancy care knowledge or even experience. Surprisingly, the spouse smoking rate was also related to less self-care. Future studies should explore the underlying reason. Our study also revealed that older pregnant women tend to report less pain/discomfort, which might be due to the tolerance to cutaneous pain increase with increasing age [36]. A correlation was also revealed between gestational age and pain/discomfort, although the underlying reason is unclear and needs to be explored in future studies.

The spouse smoking rate was related to pregnant women’s smoking status, in which the smoker group had the highest spouse smoking rate, the ex-smoker group had the second-highest spouse smoking rate, and the nonsmoker group had the lowest spouse smoking rate. This was similar to a previous study that concluded that smokers are more likely to have partners who smoke [37]. However, another study revealed that smoking exposure is associated with later depression/anxiety [38]. Following this, pregnant smokers are more likely to be exposed to both first- and second-hand smoke, and the risk of getting depressed or anxious increases even more. Therefore, local governments should advocate education of smoking cessation for both pregnant women and their families.

For ex-smokers, we found that the more cigarettes the women consumed before pregnancy, the lower their HRQoL, which is a new finding. Although a previous study explored the correlation between cigarette number and fetus health, no study has investigated the correlation between the cigarette consumption per day and the HRQoL of pregnant women [39]. Our study filled this gap.

The Banmi Online Maternity School is a free platform for all internet users and serves more than 1 million users in all the 31 provinces/municipalities across mainland China. Basically, it covers all pregnant women regardless of age, occupation, living location, past medical history, individual income, and other individual characteristics, except those who did not use the internet or pay attention to pregnancy care knowledge. Therefore, the characteristics of pregnant women in our study were not different from those of the regular pregnant women in China, except that our study did not include women who could not access the internet or did not pay attention to pregnancy care knowledge. Considering this information, the representativeness of the sample population is high. As of June 2019, the internet penetration rate in China was 61.2%, which was relatively low compared to that of South Korea and Japan, which ranged over 90% [40]. In the age of the internet, collecting information from those who do not know the internet demands a lot of human force and time and is difficult to implement. Future studies with larger groups and enough time might fill this gap.

### Strengths

This study had a large sample size, with a total of 16,483 participants from 31 provinces/municipalities across mainland China. This study filled the gap, as the effect of pre-pregnancy smoking and smoking cessation on pregnant women’s HRQoL was hardly addressed before, especially in China. This study is the first, to date, that horizontally compares pregnant Chinese women’s HRQoL among smoking-before-pregnancy, smoking-during-pregnancy, and never-smoking groups and provides statistical evidence that the more cigarettes pregnant Chinese women consume, the lower their HRQoL. This study revealed that pregnant Chinese women who stop smoking after pregnancy are more likely to suffer from depression or anxiety compared to nonsmokers.

### Conclusion

This study systematically explored the effect of the smoking period (whether before or during pregnancy), nicotine source (whether pregnant women themselves or their spouses), and the number of cigarettes consumed on the HRQoL of pregnant women. Smoking cessation during pregnancy does not significantly improve pregnant women’s HRQoL. Pre-pregnancy smoking is related to a better HRQoL (EQ-VAS score). Pre-pregnancy smoking is also related to a higher risk of anxiety/depression problems. The more cigarettes pregnant ex-smokers consume per day, the lower their HRQoL. This study provides scientific guidance for the education of pregnant women and their families about protection of both mother and baby during pregnancy. Although nicotine might benefit pregnant women’s physical health through the pain relief mechanism, its overall harmfulness for pregnant women’s HRQoL (both physical and mental health) should not be neglected. We suggest that women who have labor plans or have already conceived quit smoking and do not resume smoking and avoid an environment with nicotine, especially if their spouses or other family members smoke. However, it is common sense that quitting smoking requires a strong mind and perseverance. Therefore, for those who cannot ban smoking at home, we suggest that they separate smoking family members from pregnant women to reduce the amount of nicotine to which the pregnant women are exposed. This can be achieved by establishing a contemporary smoking room or a pregnant woman room at home.

## Abbreviations

EQ-5D: EuroQol Group’s 5-dimension
EQ-5D-5L: EuroQol Group’s 5-dimension 5-level
EQ-VAS: EuroQoL Group’s visual analog scale
HRQoL: health-related quality of life
OR: odds ratio
